# Opinion: Paradigms, methods, and the (as yet) failed striving for methodological diversity in educational psychology published research

**DOI:** 10.3389/fpsyg.2015.01370

**Published:** 2015-09-08

**Authors:** Avi Kaplan

**Affiliations:** Psychological Studies in Education, College of Education, Temple UniversityPhiladelphia, PA, USA

**Keywords:** paradigms, postpositivism, interpretivism, publishing standards, methodology in psychological research

In his March 2015 inaugural editorial, incoming *Journal of Educational Psychology* (JEP) editor Steve Graham explained his intention to build on the leadership of esteemed previous JEP editors, but also to go beyond “the status quo” to making “JEP even better” (p. 1) by setting criteria for the manuscripts that would pass the bar for peer review. Graham explicated highly commendable criteria, including adequate description of participants, and setting that would allow appropriate contextualization, replication, and generalization; demonstration of reliability and validity of measures within the context of the reported study, particularly measures of student achievement; demonstration of the fidelity of interventions, as well as description of “what happened in control and comparison conditions” (p. 2) in order to support any causal claims; and utilization of appropriate statistical analyses and report of descriptive statistics, confidence intervals, and effect sizes.

In addition to those specific criteria, Graham made another important statement highlighting the goal of enhancing JEP's methodological diversity: “Another way we plan to make JEP even better is to communicate to the field our interest in publishing high-quality research involving multiple methodologies, including quantitative, qualitative, single-subject, and mixed-methods designs” (p. 2). Enhancing methodological diversity in JEP would help to reflect the diverse nature of high-quality educational psychological research: “The world of educational psychology is very diverse in its interest and approaches to scholarship. We hope that during our watch, JEP can become even better at capturing this complexity” (p. 2). Other prominent scholars see the value of methodological diversity in published educational psychology research. More than a decade ago, then JEP incoming editor Harris (2003) made a similar call in her editorial—“We are also committed to communicating to the field our strong interest in publishing research of the highest quality across multiple methods, including qualitative, quantitative, and single-subject designs” (p. 451). Unfortunately, her call seemed to have had no apparent meaningful effect. A quick search in PsychArticles for articles in JEP with the term “qualitative” during the 12 years following Harris' editorial returned only 10 articles among hundreds, within which qualitative data were collected in six (the other four used the term qualitative theoretically), and the methodological approach was qualitative/interpretivist in only one (an article by Pressley et al., 2004). The situation appears to be only slightly different in other prominent educational psychology journals. In a thorough analysis of all empirical articles published in *Contemporary Educational Psychology* (CEP) between 1995 and 2010, Mitchell and McConnell (2012) found that only 26 (5.9%) out of the 440 articles employed qualitative or mixed-methods data. The authors concluded that “CEP remains primarily an outlet for quantitative research” (p. 140).

I strongly endorse the goal of enhancing methodological diversity in educational psychology journals. Unfortunately, I also believe that expression of interest in such diversity will not suffice to promote this desirable outcome. In this Opinion, I argue that the obstacle to methodological diversity in published educational psychology research lies in the underlying epistemological assumptions concerning what constitutes credible scientific research that has been guiding the screening standards in the leading educational psychology empirical journals. I further suggest that for promoting methodological diversity, the editors, reviewers, and, indeed, the field of educational psychology as a whole will need to engage in concerted dialogue about the legitimacy and contribution of diverse research paradigms to credible and valuable educational psychological knowledge.

I contend that a likely reason for the reluctance of educational psychology researchers who pursue research questions with designs such as case studies, life-story interviews, design-based experiments, ethnography, self-study, action research, and single-subject designs to submit their manuscripts to leading empirical educational psychology journals stems from the perception that the epistemological assumptions guiding their research are incompatible with the assumptions and corresponding standards that serve to screen out manuscripts in these journals. Qualitative data in and of themselves do not constitute an obstacle to getting published in educational psychology. In his inaugural JEP editorial, Graesser (2009) dedicated a section to qualitative research in which he noted his experience as editor of *Discourse Processes* in reviewing manuscripts that reported on studies employing qualitative data such as think-aloud protocols and naturalistic conversations. Importantly, however, Graesser explicated his preference for *how to analyze these data*: “segment verbal protocols into units, assign the units to theoretical categories, measure the number of categorized units per time period, and perform statistical tests on these quantities” (p. 261). Hence, it would be fair to assume that qualitative data, when quantified, may pass the bar of legitimacy in educational psychology. However, it is probably also fair to assume that this would not be the case for the converse—the qualitizing (Tashakkori and Teddlie, 1998) of numerical data, for example, by analyzing patterns of individuals' quantitative responses on surveys along themes similar to the way interviews are analyzed (Symonds and Gorard, 2010). Thus, rather than the type of data (qualitative or quantitative), an obstacle to promoting methodological diversity in educational psychology likely lies in editors' and reviewers' epistemological assumptions concerning the proper way to treat, analyze, and derive credible warrants from them—the “logic of justification” (Onwuegbuzie and Teddlie, 2003)—and more broadly in the research worldview, or paradigm (Kuhn, 1962), that guides the generation of research questions, the selection of methodological approach, and the derivation of inferences from findings. More specifically, I believe that the relative methodological uniformity in the leading educational psychology journals is due to the (arguably, taken-for-granted) endorsement of a paradigm that renders Null-Hypothesis Significance Testing (NHST) the ultimate approach to scientific research. This paradigm, broadly referred to as the Post-Positivist paradigm, is based on assumptions that are different from those espoused by researchers employing diverse methodological approaches.

For the purpose of illustrating differences in paradigmatic assumptions, I will briefly compare the tenets of the Post-Positivist Paradigm with another paradigm that guides social science research—the Interpretivist paradigm. The Post-Positivist paradigm is based on the ontological assumption that human experience and action are governed by general “natural” (i.e., objective) laws that operate across individuals and contexts. It further involves the epistemological assumption that while research will never be able to determine these laws absolutely, systematic testing of hypotheses about such laws among large and diverse samples and across many contexts allows for the rejection of erroneous theories and the maintenance of those that–at least for the time being—provide the best approximation to those universal laws. Importantly for educational psychological research, in the post-positivist paradigm, individual, and contextual differences are viewed as moderating factors of the operation of the universal laws. Accordingly, the randomized control trial is viewed as the most desirable study design because the randomized assignment of participants to the experimental and comparison groups is assumed to control for extraneous influences on the target phenomenon, thus allowing the attribution of any differences between the conditions to the hypothesized underlying manipulated process, leading to a warranted inference about a law of causality that can be generalized to the relevant population (for an in-depth and nuanced explication of the post-positivist paradigm, see Phillips and Burbules, 2000). Arguably, educational psychology editors, and reviewers have been regularly applying criteria derived from the Post-Positivist paradigm to evaluate any manuscript, regardless of its methodological approach.

In comparison, the Interpretivist paradigm is based on the ontological assumption that human experience and action are governed by the subjective and comprehensive meaning that people construct within the unique social-cultural contexts of their lives. It involves the epistemological assumption that research should focus on data that capture these meanings and provide insights into their content, structure, processes of formation, and consequences. In the Interpretivist paradigm, individual, and contextual characteristics are seen as inseparable from the meanings that people construct and, rather than serving as moderators, they are inherent elements of the phenomenon of interest itself. Accordingly, the optimal study designs for this paradigm are those that generate comprehensive data about people's lived experiences, the analysis of which contributes to understanding the participants' unique experiences and action as well as to insights that generalize to ever-emerging theories of human phenomena (for an explication of the Interpretivist paradigm, see Bruner, 1990).

Scholars employing the Interpretivist paradigm establish criteria to evaluate the rigor of empirical studies and the credibility of inferences (e.g., Schwandt et al., 2007). However, since these are incompatible with the Post-Positivist paradigm, even high-quality Interpretivist research is likely to be rejected from educational psychology journals such as JEP. Thus, high quality educational psychology research from the Interpretivist paradigm must seek (and does find) outlets in prestigious journals outside of Educational Psychology (e.g., *American Educational Research Journal*; *Teachers College Record*). But, this is unfortunate on many counts, primarily because the Post-Positivist and Interpretivist paradigms are not necessarily mutually exclusive. Rather, they provide alternative and potentially complementary viewpoints from which to ask different research questions about phenomena (Maxwell, 2004). For example, generalized principles of human functioning may provide starting points for in-depth investigation of and intervention in people's meaning construction in specific social-cultural contexts, and these investigations, in turn, may lead to insights about the way those general principles operate in the complexity of human life. Incorporating knowledge from research employing these paradigms, as well as other paradigms that highlight cultural-historical processes (Wertsch, 1991), power relations (Teo, 2015), and the dynamics of complex systems (Guastello et al., 2009), may be the only viable way to gain comprehensive understanding of the complex and multi-faceted phenomena that are of interest to educational psychologists.

However, currently, the educational psychology community seems to be doing little to encourage learning and deliberating about the legitimacy of different research paradigms. Arguably, formal teaching of the philosophical underpinning of different research paradigms in our doctoral programs is rare, and students are commonly socialized to endorse Post-Positivist assumptions and to apply the NHST as the single legitimate scientific approach. Unfortunately, this may have been one reason for the perception that educational psychological research is irrelevant to educational practice (Berliner, 2006). I believe that educational psychology and its flagship journals must not stay singly entrenched in the NHST approach at this critical moment in time –a time when the American Psychological Association embraces the Society for Qualitative Inquiry in Psychology (SQIP) and launches the new journal *Qualitative Psychology*, when the Carnegie Foundation for the Advancement of Teaching adopts the contextualized practice-oriented “Improvement Science” approach as its paradigm for educational research and interventions (Carnegie, 2015)^1^, when the Institute for Educational Sciences (IES) initiates funding for partnership programs under a Continuous Improvement Research in Education (CIRE; IES, 2015^2^), and when Design-Based Implementation Research (DBIR, Fishman et al., 2013) is gaining clout as the viable approach for interdisciplinary interventions in pertinent educational issues. It is imperative that the educational psychology community engage in a concerted dialogue about the different paradigms that could frame educational psychological research and the criteria for high-quality investigations within each of these paradigms, that the training of educational psychology doctoral students involve engagement in philosophy of science and the exploration of different logics of justification (Eisenhart and DeHaan, 2005), and that the editorial teams and boards of top educational psychology journals incorporate scholars who are able to apply the relevant ontological, epistemological, and methodological assumptions to evaluate the merit of manuscripts reporting on studies with diverse methodological approaches.

## Conflict of interest statement

The author declares that the research was conducted in the absence of any commercial or financial relationships that could be construed as a potential conflict of interest.

## Footnotes

^1^Carnegie. (2015). Available online at: http://www.carnegiefoundation.org/our-ideas/six-core-principles-improvement. [Accessed on April 25, 2015].

^2^IES. (2015). Available online at: http://ies.ed.gov/funding/ncer_rfas/cir.asp. [Accessed on April 25, 2015].
